# Comments on recent community water fluoridation studies

**DOI:** 10.1038/s41415-023-6338-z

**Published:** 2023-10-27

**Authors:** Simon Hearnshaw, Barry Cockcroft, Andrew Rugg-Gunn, A. John Morris, Raymond J. Lowry, John Beal, Johnny Johnson, Matt Jacob

**Affiliations:** 41415368537001 UK National Community Water Fluoridation Network, Leeds, UK; 41415368537002 British Fluoridation Society, Oldham, UK; 41415368537003 University of Birmingham, Birmingham, UK; 41415368537004 American Fluoridation Society, Portland, Oregon, USA

## Abstract

Water fluoridation is a public health measure to reduce levels of dental caries in populations. A report of the recently completed CATFISH study has been published. This was the first UK evaluation of fluoridation introduction for many years; it was carefully designed and executed and is welcomed. The purpose of this article is to highlight the 180-page report of the study and comment on some aspects to aid interpretation of the findings. Significant features were that two cohorts, from birth and from five years, were followed for six years in a fluoridated and a non-fluoridated area, and clinical and cost analyses were reported. Areas of the report which deserve comment are: a) interruption of fluoridation for a year for half the children in the intervention area and its effect; b) clinical results were reported as absolute reduction in caries prevalence, with the preventive fraction for caries severity downplayed; c) the power of the study was diminished by an unexpected lower caries increment; and d) control for differences in diet was not possible. Nevertheless, this major UK study showed clinical and cost benefits of water fluoridation. The risk of cessation of water fluoridation is highlighted in examples of three recently published studies.

## Key points

- The recently published report of the CATFISH study is welcomed, showing the clinical and cost benefits of water fluoridation.
- Comments are made on some aspects of the report to aid understanding.
- The adverse effect of cessation of water fluoridation is highlighted in three recently published studies.

## Introduction

The first large-scale evaluation of the introduction of water fluoridation for many years in the UK has recently been published. As dental health leaders and consultants in the UK and USA, we have been encouraged by colleagues to offer our assessment of this study known as CATFISH (Cumbria Assessment of Teeth - a Fluoride Intervention Study for Health).^1^ CATFISH was funded by the National Institute of Health and Care Research.

Water fluoridation is the adjustment of fluoride concentration in water supplies to the optimal concentration for reducing caries. In a collective statement, the chief medical officers^2^ of England, Northern Ireland, Scotland and Wales cited the 'strong scientific evidence' of fluoridation's ability to reduce decay and promote dental health equality. Fluoridation has wide international support, including the Centres for Disease Control and Prevention^3^ in the USA, the governments of Canada^4^ and Australia,^5^ and the Irish Expert Body on Fluorides and Health,^6^ as well as the World Health Organisation.^7^

A 2015 Cochrane systematic review found that because of the introduction of fluoridation, children suffered 35% fewer decayed, missing and filled primary teeth (dmft) and 26% fewer decayed, missing and filled permanent teeth (DMFT).^8^ There were, however, reservations about the contemporary impact, since many studies pre-dated the widespread introduction of fluoride toothpaste.

An additional way to confirm the benefits of fluoridation is to study the impact after a community has ceased this practice. The above Cochrane review^8^ said there was 'insufficient information to determine the effect of stopping water fluoridation programmes on [tooth decay]'. However, since this assessment, three new studies from Canada, United States and Israel have been published analysing the impact of cessation, and their findings will be summarised.

## The CATFISH findings

The CATFISH study was conducted to meet the inclusion criteria stipulated by the York systematic review addressing the design issues identified by the Medical Research Council.^9^

CATFISH examined the effects of a fluoridation scheme in the North West England county of Cumbria. The study looked at two groups: a birth group of children born after fluoridation was introduced, and an older group who were around five years of age at the start of the research project. CATFISH compared dental health across the intervention and control groups over five to six years, monitoring the dental health of a sample of children in West Cumbria where fluoridation was reintroduced in 2013, and a sample of children across the rest of Cumbria which remained unfluoridated throughout the study period.

Dental teams conducted examinations at regular intervals, taking photographs of teeth which were blinded for examiners' analysis, and researchers conducted questionnaires and collected information on social deprivation, brushing and diet. At the end of the study, 1,444 five-year-olds who were part of the younger cohort and 1,192 eleven-year-olds who were part of the older cohort had taken part.

CATFISH is welcomed as a well-designed contemporary water fluoridation study. Much like other international studies, the CATFISH research revealed that children living in fluoridated areas of Cumbria had better dental health. The primary outcome analysed by the CATFISH researchers was whether any tooth decay was present or not in a child's mouth. The study reported 'a modest beneficial effect' for children living in fluoridated areas. This modest benefit, the authors wrote, 'needs to be considered against the use of other dental caries preventative measures'.

As with most research, there are limitations that may conflict the study outcomes. Several limitations are documented within the report. The following limitations are worthy of deeper analysis to understand how they might have affected the study's outcomes.

### Fluoridation interruption

The most serious limitation of the study is that more than half of the children in the fluoridation group experienced a one-year interruption to fluoridation as the essential equipment serving the Ennerdale area was not operational in 2015/16 due to flooding. Simply put, children were not receiving an optimal amount of fluoride as is normally intended through fluoridation and in fact were receiving zero fluoridation for a sustained period. This is a significant limitation and cannot be described as 'normal' expression of fluctuation in dosing.

If a randomised trial were testing the impact of fluoride toothpaste and it turned out that a portion of the intervention group unexpectedly received toothpaste without fluoride, this would understandably be viewed as casting a cloud over the resulting outcomes. This is essentially what unfortunately transpired in CATFISH about which researchers had no control.

*Post hoc* analyses showed that caries prevention was greater in children who received continuous water fluoridation compared with children who received interrupted water fluoridation - details are given below.

### Downplaying significant differences

CATFISH uses presence/absence of decay as the primary outcome, describing (in the authors' words) 'modest' reductions in tooth decay. However, previous studies typically express effect as the preventive fraction, for example, this Cochrane systematic review on fluoride toothpastes.^10^ For the primary outcome of caries prevalence, differences between the intervention and control groups were expressed in the report as absolute differences of 4% and 3%. If the differences are expressed as the preventive fraction (PF), the differences are 18.7% improvement in the birth cohort and 12.8% in the five-year-old cohort. These PF percentages cast the outcome in a new light.

D_3_MFT/d_3_mft is quoted as a secondary outcome but not decayed, missing and filled surfaces, which may describe the impact of fluoridation better. For children with most decay, there was a 36% reduction in the proportion of children with four or more dmft in the fluoridated areas.

In both the birth and five-year-old cohorts, there was a significant disease reduction across dmft/DMFT. Mean dmft in the birth cohort was 0.49 in the intervention group and 0.69 in the control group - an absolute difference of 0.2 dmft and a PF of 29% (similar to PFs reported in other recent water fluoridation studies).^5^^,^^6^^,^^8^ This means that dental decay experience was 29% lower for young children drinking fluoridated water. For the older children, who received water fluoridation from five years, the PF was 20%.

For the birth cohort, the odds ratio - a measure of the likelihood for developing decay - for children living in the non-fluoridated area compared with children living in the fluoridated area was 1.36 (95% CI: 1.02-1.81). However, for children from the most deprived quintile, the odds ratio was higher at 2.18 (95% CI: 1.23-3.90). Although we understand why the CATFISH authors highlighted the primary outcome (prevalence), the significant differences in the severity of decay are perhaps more important, for example, from a treatment viewpoint. Citing the absolute differences rather than PFs has the effect of downplaying the dental health benefits for children in the fluoridated areas.

### Study size

The sample sizes for the CATFISH study were based around estimated relatively high decay rates of around 40%. Reduced decay prevalence impacts adversely on the power of the study. Given that the numbers completing the five-year study were only slightly above the numbers estimated to be required, and that development of decay was substantially less than expected, the power of the study to detect a difference (risk ratio [RR] of 0.8) is questionable.

### Insufficient statistical power

The power of a study represents the probability of finding a difference that exists within a population. Low power means that an analysis is much less likely to detect an actual effect or that results are distorted by systematic or random error. The authors do present a *post hoc* analysis for the birth cohort which had continuous exposure and which showed an 8.1 percentage point difference in caries prevalence (d_3_mft >0) between exposure and control groups (RR: 0.62; 95% CI: 0.43-0.89). However, the study was not powered to analyse these differences.

Likewise, an analysis for the older cohort which received consistent fluoridation is presented in the report showing a 3.7 percentage point difference in caries prevalence (D_3_MFT >0) between exposure and control groups (RR: 0.83; 95% CI: 0.59-1.18). But, as for the birth cohort, the study lacks power for this analysis. In the results, the more consistent fluoridation intervention results are merged with the results from the Ennerdale group which received significantly interrupted fluoridation.

### Balance of intervention and control groups

Controlling balance is always difficult across the intervention and control populations within any large study. In the CATFISH study, there were significant differences across deprivation, where the intervention groups had a greater proportion of children from the most deprived quintile (30% in the intervention group compared with 20% in the control group). Additionally, the intervention group reported more cariogenic diets and poorer dental attendance than the non-fluoridated population. These factors mean that the preventive effect of fluoridation was being examined within population groups with greater disease risk compared to the control groups.

### Cost effectiveness

CATFISH reported that for the birth cohort, there is a 77.0% probability of fluoridation being cost-effective, and for the older cohort, there is a 68.3% probability of it being cost-effective. These probabilities might have been even higher had the study considered the full potential of water fluoridation, often referred to as the 'halo effect' (see next section).Additionally, when the CATFISH authors suggest comparing the benefits of fluoridation with other preventive measures, it is worth considering the conclusions of the Israel study discussed later, showing that fluoridation significantly reduced decay-related treatment needs while a free dental care programme did not.

Besides comparing the benefits of preventive measures, their costs should also be compared. Such a comparison is critical to ensure that the most cost-effective measures are prioritised. In this regard, it is noteworthy that the *per capita *cost of fluoridation is lower than supervised toothbrushing schemes and perhaps every other large-scale preventive programme.

### Halo effect

One limitation not described in the report is the potential 'halo effect'. This refers to the way in which people outside of fluoridated areas can benefit from such a scheme because food and beverages produced with fluoridated water are transported to and consumed in non-fluoridated areas. *Vice versa,* the halo effect also describes how food and beverages made in adjacent non-fluoridated areas are consumed in an adjacent fluoridated area. This is particularly the case where the areas are small and where the intervention and control groups are geographically alongside each other. The halo effect within the CATFISH study could have reduced the outcome difference.

## Non-UK studies of the effect of cessation of water fluoridation programmes

### Canada

Over a 14-year period, researchers compared tooth decay trends among children in Edmonton, a continuously fluoridated city, with those in Calgary, which ceased water fluoridation. The most recent analysis was published in 2021. Initially, the proportion of second grade (7-8-year-old) children in Calgary with at least one decayed, extracted or filled tooth (deft) was lower than the proportion among Edmonton children. The decay in primary teeth in Calgary began to rise by 2010, and this increase accelerated after cessation in 2011. By the end of the 14-year study period, the deft rate for Edmonton children stood at 55%, which is approximately where it was at the start of the study. By contrast, Calgary's deft rose from about 49% to 65%.^11^

### USA

A 2022 study in the US state of Alaska compared the costs of decay-related dental treatments for children from deprived neighbourhoods in two cities. In Anchorage, which was fluoridated throughout the nine-year study period, the cost of treating children's decay rose by only 5% between 2003 and 2012. In Juneau, where fluoridation had ceased in 2007, decay-related costs increased by 47%.^12^

### Israel

Nationwide fluoridation had been instituted in Israel in 2002, and this resulted in declines in tooth decay. But a legislative change in 2014 discontinued this practice. Free dental services for children and adolescents were promoted as an alternative way to reduce tooth decay. In a 2023 study, researchers analysed dental records of more than 34,000 adults. They concluded that Israel's fluoridation law was linked to 'significantly lower caries-related treatment needs while national dental health legislation providing free dental care to children and adolescents was not'.^13^

## Conclusion

Overall, the CATFISH study adds to the body of evidence supporting the view that fluoridation improves dental health and is a cost-effective intervention. It is unrealistic to expect all studies of the same intervention to reveal an identical effect on a population.

The recent non-UK studies report a greater impact on reducing the incidence and cost of tooth decay than the CATFISH study. Interestingly, the seemingly 'modest' benefits reported by CATFISH would be perceived as much more compelling if the authors had calculated and reported the PF differences, for example, the 36% reduction in the proportion of children with four or more dmft.

As the CATFISH study states, 'a single study cannot hope to provide definitive answers'; however, it can add to the existing evidence base within the context of the study's limitations. Dental practitioners, public health officials and health policymakers should recognise the strengths and limitations of CATFISH. Accordingly, they should be aware of the findings from the recent Canadian, American and Israeli studies on the impact of ceasing fluoridation. By doing so, they can offer appropriate guidance to patients and adopt evidence-based policies.
